# Patients as team members: Factors affecting involvement in treatment decisions from the perspective of patients with a chronic condition

**DOI:** 10.1111/hex.13358

**Published:** 2021-10-01

**Authors:** Martina Buljac‐Samardzic, Mark A. Clark, N. Job. A. van Exel, Jeroen D. H. van Wijngaarden

**Affiliations:** ^1^ Department Health Services Management and Organisation, Erasmus School of Health Policy and Management Erasmus University Rotterdam Rotterdam The Netherlands; ^2^ Kogod School of Business American University Washington DC USA; ^3^ Department Health Economics, Erasmus School of Health Policy and Management Erasmus University Rotterdam Rotterdam The Netherlands

**Keywords:** chronic disease, patient‐centred care, patient involvement, patient perspective, Q‐methodology, the Netherlands

## Abstract

**Background:**

Active patient involvement in treatment decisions is seen as a feature of patient‐centred care that will ultimately lead to better healthcare services and patient outcomes. Although many factors have been identified that influence patient involvement in treatment decisions, little is known about the different views that patients have on which factors are most important.

**Objective:**

This study explores the views of patients with a chronic condition on factors influencing their involvement in treatment decisions.

**Design:**

Q‐methodology was used to study the views of patients. Respondents were asked to rank a set of 42 statements from the least important to the most important for active patient involvement in treatment decision‐making. The set of 42 statements was developed based on a literature search and a pilot in which two external researchers, 15 patients and four healthcare professionals participated. A total of 136 patients with one of three major chronic conditions were included: diabetes types 1 and 2, respiratory disease (i.e., chronic obstructive pulmonary disease and asthma) and cancer (i.e., breast cancer and prostate cancer). Data were collected in a face‐to‐face interview setting in the Netherlands.

**Results:**

Four distinct views on the factors influencing active patient involvement were identified among patients with a chronic condition. (1) Enabled involvement: the extent to which patients are facilitated and empowered to participate will lead to patient involvement. (2) Relationship‐driven involvement: the relationship between patients and healthcare professionals drives patient involvement. (3) Disease impact‐driven involvement: the severity of disease drives patient involvement. (4) Cognition‐driven involvement: knowledge and information drive patient involvement.

**Discussion and Conclusion:**

From the patients' perspective, this study shows that there is no one‐size‐fits‐all approach to involving patients more actively in their healthcare journey. Strategies aiming to enhance active patient involvement among patients with a chronic condition should consider this diversity in perspectives among these patients.

**Patient Contribution:**

Patients are the respondents as this study researches their perspective on factors influencing patient involvement. In addition, patients were involved in pilot‐testing the statement set.

## 1 INTRODUCTION

Active patient involvement has been high on the agenda in recent decades and is seen as an important feature for patient‐centred care. Patient involvement can take many forms, for example, by involving patients in governmental policies on healthcare, research or hospital policy.^1^ Nowadays, patients are especially expected to be partners of healthcare professionals in the treatment decision‐making process, as their experiential knowledge is seen as complementary to professionals' knowledge.^2^, ^3^ It is increasingly being recognized that patient involvement in decision‐making can improve medical outcomes, patient satisfaction and quality and safety of care.^4^, ^5^, ^6^, ^7^, ^8^, ^9^, ^10^, ^11^ Many studies have therefore focused on identifying factors that may enhance patient involvement in treatment decision‐making.^12^, ^13^ A wide range of studies have assessed factors that act as facilitators and barriers to patient involvement in treatment decisions. Next to the more general literature on patient involvement in treatment decision‐making,^14^ there is also literature that focuses on patient involvement within a specific disease group^15^, ^16^, ^17^, ^18^ or with respect to a certain outcome such as patient safety.^5^, ^7^ In addition, relevant factors can be found in the literature on shared leadership and teamwork between healthcare professionals, informal caregivers and patients, in which patients are considered as team members in terms of their involvement in treatment decisions.^19^, ^20^ Due to the fast‐growing literature and the overwhelming number of identified influencing factors, it is currently difficult to paint a clear picture on what matters the most for patient involvement in treatment decision‐making. Furthermore, people who participate in the care process seem to have different views on patient involvement. Research has revealed differences between the points of view of patients, informal caregivers and healthcare professionals, but also showed variety within these groups.^21^, ^22^, ^23^ From the healthcare professionals' perspective, different studies have shown divergent views on their roles in self‐management support.^24^, ^25^, ^26^ For example, van Hooft et al.^24^ distinguished four perspectives on the goals for self‐management support among nurses (i.e., the coach, the clinician, the gatekeeper and the educator perspective).^24^ Been‐Dahmen et al.^25^ identified three divergent views among nurses on patient and nurse roles in self‐management support (i.e., adhering to a medical regimen; monitoring symptoms; and integrating illness into daily life).^25^ Also, Aasen et al.^26^ identified three discursive practices among nurses on the participation of patients and their relatives in end‐of‐life decisions (i.e., the nurses' power and control, sharing power with the patient and transferring power to the next of kin).* From the patient perspective*, available studies often present the view of a specific group of patients. Jedeloo et al.^27^ identified different preference profiles for healthcare delivery and self‐management among adolescents with chronic conditions (i.e., conscious and compliant, backseat patient, self‐confident and autonomous and worried and insecure). O'Brien et al.^23^ identified facilitators and barriers to patient involvement from the perspective of patients with early‐stage breast cancer and their physicians. Although the variance in patient preferences to participate in treatment decisions is acknowledged,^7^, ^28^ there is a widespread belief that most patients wish to be involved in decision‐making at least to some extent, either in terms of shared decision‐making with their physicians and/or informal caregivers or by making their own decisions.^29^, ^30^

Considering the number of factors that may influence active patient involvement identified in previous literature, but also acknowledging that preferences may differ between patients, it seems important to understand the patient perspective on these factors better. Therefore, the aim of the present study is to explore the diversity of views among patients on factors influencing their involvement in treatment decisions.

## 2 METHODOLOGY

### 2.1 Setting

Studying patient perspectives on what is important for active involvement seems especially interesting for patient groups who could gain the most from involvement and have the most opportunities to be involved.^31^ Chronically ill patients, who undergo long‐term treatment, have to adjust to changes in their condition and/or their treatment over time and have long‐lasting relationships with healthcare professionals are therefore the most suitable target group for the present study.^31^ To study the views of patients with chronic diseases, we selected patients from three major, distinct types of chronic diseases: diabetes, chronic obstructive pulmonary disease (COPD)/asthma and cancer. Then, we looked at possible relevant subdivisions within these groups related to differences in care trajectories. We decided to include both type 1 and type 2 diabetes patients, and both COPD and asthma patients. As there are many types of cancers, we decided to choose the two most common invasive forms of cancer for both men and women: prostate cancer and breast cancer. A Q‐study usually has a sample size between 30 and 40 respondents,^32^, ^33^ but to capture the variety in views in this diverse sample, we decided to increase the sample size. We recruited around 20 patients from each (sub) type. The final study sample consisted of 136 respondents: 19 diabetic type 1 patients, 21 diabetic type 2 patients, 26 COPD patients, 25 asthma patients, 23 breast cancer patients and 22 prostate cancer patients. All of the respondents were living at home and received care from formal and informal caregivers. Depending on the severity of their condition (at that time), they interacted with primary care (GP and often a nurse practitioner) and/or secondary care (mostly a medical specialist from a general hospital). In case of asthma and diabetes, for example, respondents interacted most of the time with their primary care professionals, as is customary in the Dutch healthcare system.

### 2.2 Q‐methodology

We used Q‐methodology to explore and compare the perspectives of patients on active involvement in their treatment decisions. Q‐methodology is frequently used to study the views, attitudes or perspectives of patients, professionals and other stakeholders in healthcare research, for example, views on medical leadership, patient‐centred care, vaccination and effective teams.^34^, ^35^, ^36^, ^37^, ^38^ Q‐methodology is particularly useful for the purpose of this study because it combines aspects of qualitative and quantitative methods for an in‐depth study of potentially complex and diverse subjective topics, such as patient perspectives.^39^, ^40^ The core of the data collection consists of respondents reading, evaluating and ranking a broad set of statements about the topic, usually between 40 and 60 statements, according to their perspective on the topic. The combination of this elaborate ranking exercise that is the same for all respondents and a follow‐up interview that delves deeper into the individual perspective of respondents that they reveal through their ranking of the statements results in a rich data set, minimizing researcher bias and allowing respondents' voices to be heard in a unique way.^41^, ^42^, ^43^

The study was conducted in four stages: (1) development of the list of factors that influence patient involvement (statement set); (2) selection of respondents; (3) data collection; and (4) data analysis. Because this study seeks to explore patient perspectives rather than confirm particular theoretical constructs, no formal hypotheses were formulated. However, we note that the development of the statement set is guided by existing research identifying factors likely to affect patient perspectives.

#### 2.2.1 Development of the statement set

The statement set should broadly cover the variety of factors that may influence active patient involvement. First, the literature was reviewed to identify the main factors related to patient involvement in treatment decision‐making. In our endeavour to develop a comprehensive list of factors, we drew upon literature on patient involvement (and related concepts such as self‐management and shared decision‐making), shared leadership and teamwork. On the one hand, we studied literature reviews.^5^, ^7^, ^15^, ^17^, ^44^ It is noteworthy that Dwarswaard et al.^13^ performed a literature review on self‐management support from the perspective of patients. On the other hand, we selected studies that investigated factors influencing patient involvement based on empirical data (both qualitative and quantitative designs), some from the perspective of healthcare professionals and^45^ others from the patient perspective.^45^, ^46^, ^47^, ^48^ Synthesizing these studies, we identified five general categories of factors influencing the ability, motivation and opportunity of patients to be involved in treatment decision‐making: patient‐related factors, healthcare professional‐related factors, patient–professional interaction‐related factors, contextual factors and illness‐related factors. *Patient‐related factors* refer to the individual characteristics and experiences of patients such as their coping style, ability to access, understand and use information (health literacy) and past experiences with health and social care and formal care providers. The presence of and interaction with the patient's personal support network are an important element of this first category.^5^, ^15^, ^17^, ^48^ *Healthcare professional‐related factors* refer to the individual characteristics (e.g., gender, ethnicity) and attitudes of care providers (e.g., dependency towards a patient).^5^, ^44^, ^45^ *Patient‐professional interaction‐related factors* include trust, appreciation, encouragement between patients and professionals and the interaction among healthcare professionals.^5^, ^15^, ^17^, ^44^, ^46^ *Contextual factors* refer mostly to the organization of care and the availability of resources, such as the available time for consultations, available information, availability of materials and the use of technology to support patients.^15^, ^44^, ^48^ *Illness‐related factors* reflect the prospective course, the stage, the severity and the impact of the illness, but also the presence of different treatment options and acceptable alternatives.^5^, ^15^, ^17^

Next, in keeping with this, a comprehensive list of statements was compiled by the authors that aimed to cover the content of these five factors and this initial set of 60 statements was then pilot‐tested. To assess content validity, two external researchers with expertise in patient involvement were consulted. To assess face validity, 15 patients (i.e., two diabetic type 1 patients, three diabetic type 2 patients, two COPD patients, two asthma patients, two breast cancer patients, four prostate cancer patients) and four healthcare professionals engaged with patient involvement were consulted (i.e., one physician, three paramedics). In an interview setting, they were asked to rank the statements and reflect on them by judging the clarity of the statements and the completeness of the set. Based on this pilot test, 10 statements were deleted and eight statements were combined with other statements. This reduced the statement set to 42 statements. Thereby, multiple statements were rephrased from minor to major revision, for example, from ‘how well a patient knows the healthcare system’ to ‘how well a patient knows where he/she can go with a request for help’ or from ‘how well a patient mastered the spoken and written Dutch language’ to ‘how well a patient mastered the Dutch language’. The final statement set consisted of 42 statements covering patient‐, motivation‐ and opportunity‐related factors in terms of active patient involvement (see Table 1).

**Table 1:** Statement set and factor scores

S. no.	Statements	Factor arrays			
		View 1	View 2	View 3	View 4
1.	How physically healthy a patient feels	2*	0*	3*	2*
2.	How mentally healthy a patient feels	2*	−1*	1*	−3*
3.	How much a patient knows about this disease	−1	−1	2*	4*
4.	How much experience a patient has with this disease	−2*	−1*	2*	0*
5.	Experience of a patient with active participation in the treatment decision process	−2**	−1	0*	‐1
6.	Negative experience of a patient in dealing with caregivers	−1	0	−1	−2
7.	Positive experience of a patient in dealing/interacting with healthcare providers	1*	2*	−1	−1
8.	How well a patient understands information about his/her own health	4	2	1	3
9.	How well a patient can find information about this disease	0	−2*	1	2
10.	How well a patient can have a conversation with his/her healthcare provider	1	3*	2	2**
11.	How well a patient knows where he/she can go with a request for help	2*	2*	0	0
12.	The way a patient deals with setbacks	1*	−1	0	0
13.	The intelligence of a patient	−3	−2*	3*	−3
14.	How well a patient mastered the Dutch language	0	1*	−1	−3*
15.	Patient's preference to actively participate in the treatment decision process	0	1**	2**	0
16.	The extent to which a patient feels taken seriously by his/her caregivers	0	4*	0	3*
17.	How much a patient trusts his/her caregivers	4*	4*	1	1
18.	A patient thinks that his/her active participation has advantages	0**	1*	3*	−1**
19.	Heard about active participation from other patients	−3	−3	−1	−2
20.	Patient's fear of medical treatment	1*	−2*	−1	−1
21.	The effect of the disease on the daily functioning of a patient	1**	1**	4*	3**
22.	The future expectations of this disease for a patient	3*	0**	0**	2*
23.	Having care providers who encourage active participation	−1*	3*	1	1
24.	Having care providers who realize that they are dependent on the patient	−4*	0*	−3*	1*
25.	Having family and friends who support a patient with active participation	3*	0	0	0
26.	Having care providers who show appreciation for active participation	0*	2	1	−2*
27.	To what extent a patient dares to give his/her opinion	1	3	2	0*
28.	Receiving care from a healthcare organization	−1	0*	−2	−1*
29.	Financial consequences of care for a patient	−2	−2	−2*	−1
30.	The available time of a patient	−2**	−3*	0**	−1**
31.	Having a choice of multiple equivalent treatment options	2**	0**	−1**	1**
32.	The severity of the disease	3*	1*	4*	4*
33.	Having a care provider of the same gender as the patient	−4**	−4**	−4	−4
34.	Having a care provider of the same ethnicity as the patient	−3	−4	−4	−4
35.	How well different care providers work together	0	1**	−3*	1
36.	Availability of all relevant information	1	‐2*	1	1
37.	The time pressure that a patient experiences during a consultation	0	0	−2	−2
38.	The accessibility of one's own medical data	−1	−1	−1	2*
39.	Maintaining the same healthcare providers	−1	2*	−3*	‐2
40.	Possibilities to adjust the care to the needs of a patient	2*	1	0*	1
41.	Availability of technical aids to support active participation of a patient	−1	−1	−2*	0**
42.	Online support for active participation of a patient	−2	−3*	−2	0*

*Note*: There were no consensus statements, statements that do not distinguish between any pair of factors.

* Distinguishing statements for a view (*p* < .01).

** Distinguishing statements for a view (*p* < .05).

#### 2.2.2 Selection of respondents

As there was no previous literature to inform us about the number of views on active involvement in treatment decisions among patients with a chronic condition, or which characteristics of patients might be related to holding particular views, we aimed for a diverse sample of patients, varying in age, gender and type of condition. We recruited respondents through convenience sampling via personal and professional networks and social media. Each participant was asked for informed consent, with the guarantee that data would be anonymized before publication. A total of 136 patients from three distinct chronic patient groups (i.e., diabetes, respiratory disease and cancer) across multiple hospitals in the Netherlands were included. For the purpose of this study, which is to identify the diversity of views among patients, this is a relatively large sample, as each participant performs a large number of tests by relating all statements to each other.^39^, ^42^ Determining the prevalence of the views in a larger patient population or their association with respondent characteristics is not the purpose of this study; applying survey methods and representative subject sampling would be more appropriate for this purpose.

#### 2.2.3 Data collection

**Figure 1:**
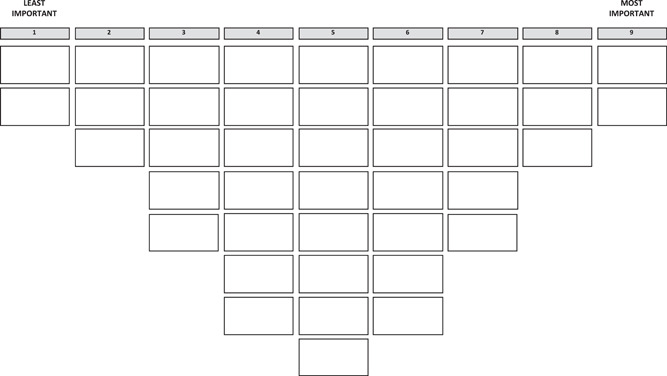
Sorting grid that respondents used to rank the 42 statements from the least important to the most important for active patient involvement

In a face‐to‐face interview setting in the Netherlands, respondents were asked to rank 42 statements from the least important to the most important for active patient involvement in treatment decisions using a sorting grid (Figure 1). These one‐on‐one sessions between the patient and the researcher were recorded, summarized and translated from Dutch to English. The data collection consisted of three steps and was conducted before the coronavirus disease pandemic of 2019. In the first step, respondents were instructed to first read all the statements; each statement was printed on a separate card. While reading each statement, the respondents were asked to divide the cards into three piles: important for patient active involvement, unimportant for active patient involvement and neutral. In the second step, respondents were instructed to read all the statements placed on the ‘important pile’ once again and to select the two most important statements, and then place them in the two spots on the extreme right side of the sorting grid (under 9). From the remaining statements in the ‘important pile’, respondents then selected the three statements that they found most important and placed these on the sorting grid (under 8), and so on until there were no statements left in the ‘important pile’. This was repeated for the ‘unimportant pile’, which was ranked from the left side of the sorting grid, followed by the ‘neutral pile’, which was ranked in the remaining spots in the middle of the sorting grid. This resulted in a completely filled sorting grid. In the third step, respondents were asked to motivate their ranking of the statements. Respondents were asked to explain the reasoning behind the placement of the two statements they considered most important and then the reasoning for the two least important statements. In addition, respondents were asked to answer a number of questions about personal characteristics (i.e., gender, age, living situation, hours of informal care per week and relation to informal caregiver, years of experience with the disease and formal care). Respondents were also asked to rate their health status on a paper‐based Visual Analogue Scale, which is a vertical line that represents a continuum from 0 (*worst conceivable health*) to 100 (*best conceivable health*).

#### 2.2.4 Data analysis

Analyses were conducted using the PQMethod 2.35 software package.^49^ First, a correlation matrix between the rankings of the statements by respondents was computed to inspect the degree of similarity between the rankings. Second, the correlation matrix was subject to a by‐person factor analysis (centroid factor analysis with a varimax rotation) to identify groupings of respondents who ranked the statements in a similar way. Finally, weighted average rankings of the statements were computed for each resulting factor and three researchers independently interpreted these statistical results as distinct views on what is important for active patient involvement. Consensus on the number of factors to retain and their preliminary interpretation was reached through debate. The interpretation of the chosen set of factors was finalized using the qualitative data obtained from respondents for each factor focusing on the motivations provided by the respondents who were statistically significantly associated with that factor.

#### 2.2.5 Ethical considerations

An appropriate medical ethical committee judged that this study did not require formal ethical approval under Dutch law because it does not concern ‘medical/scientific research’ about illness and health, nor did the content or methods cause ‘an infringement of the physical and/or psychological integrity’ of the participants (MEC‐2017‐318; WT/sl/318713). Respondents were assured of the confidentiality of their responses and provided their written consent to use the data they provided for the purpose of this study.

## 3 RESULTS

### 3.1 Respondents' characteristics

The study sample consisted of 136 respondents: 19 diabetic type 1 patients, 21 diabetic type 2 patients, 26 COPD patients, 25 asthma patients, 23 breast cancer patients and 22 prostate cancer patients. The mean age of the respondents was 50.7 years, and 53.7% were female. The majority of the respondents (72.1%) were living with their informal caregiver, half of the informal caregivers (50%) were the patients' partners and informal caregivers provided on average 12 h of care per week. Respondents were in relatively good health, scoring 70.8 on a Visual Analogue Scale ranging from 0 (*worst conceivable health*) to 100 (*best conceivable health*). The perceived health status varied between 30 and 95 in this study sample. Table 2 presents an overview of the respondents' characteristics.

**Table 2:** Respondents' characteristics

	*n*	%	
Type of disease			
Diabetic type 1	19	14.0	
Diabetic type 2	21	15.4	
COPD	26	19.1	
Asthma	25	18.4	
Breast cancer	23	16.9	
Prostate cancer	22	16.2	
Gender			
Male	63	46.3	
Female	73	537	
Living situation			
Alone	38	27.9	
With parents	8	5.9	
With relative(s)	3	2.2	
With a partner	45	33.1	
With partner and child(ren)	31	22.8	
Other	11	8.1	
Informal caregiver^a^			
Partner	68	50.0	
Child(ren)	28	24.8	
Parent(s)	29	21.3	
Relative(s)	33	24.3	
Neighbour(s)	8	5.9	
Friend(s)	34	25.0	
	Mean	SD	
Years with disease	12.9	12.8	
Years with formal care	11.7	12.4	
Hours of informal care per week	12.0	32.2	
	Mean	SD	Min–Max
Age	50.7	20.3	16–86
Perceived health status (VAS 1‐100)	70.8	12.2	30–95

Abbreviation: COPD, chronic obstructive pulmonary disease.

^a^ Note that the percentages do not add up to 100% as respondents can have multiple caregivers.

### 3.2 Four views on what is important for active patient involvement

**Figure 2:**
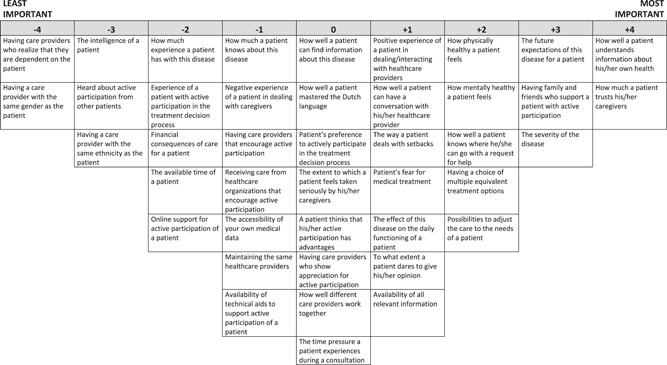
Ranking of 42 statements from the least important to the most important for view 1: enabled involvement

The analysis revealed four views among patients with a chronic disease on what is important for them to act as a team member in their healthcare journey, as outlined below. Table 1 presents per view the ranking of statements from the least important to the most important. Figure 2 visualizes the ranking for the first view.

### 3.3 View 1: Enabled involvement

This view is about patients being able to participate, through the support of family and friends (Statement 25, +3) as ‘everybody needs help’ and ‘It's important to be able to rely on your family and friends’ (Respondent P5). Also, by understanding information about their own health (Statement 8, +4) and trusting their healthcare professionals (Statement 17, +4), these respondents emphasize that it is about facilitating and enabling, not about informal carers deciding for patients, because ‘being able to function reasonably independent is important’ (Respondent P5). In addition, the severity of the disease (Statement 32, +3) and the future expectations for the patient (Statement 22, +3) are important for patient involvement because ‘If there are no other treatment options, then it does not make sense to be actively involved in healthcare decision‐making’ (Respondent B2). In line with this, physical (Statement 1, +2) and mental (Statement 2, +2) health are seen as relatively important. It is notable that enabling patient involvement through an online platform is seen as relatively unimportant (Statement 42, −2). Also unimportant are factors that relate if professionals realize that they depend on the patient to provide good treatments (Statement 24, −4) and factors that relate to what extent the patient and the healthcare professionals are familiar with each other, have the same ethnic background (Statement 34, −3) or are of the same gender (Statement 33, −4).

### 3.4 View 2: Relationship‐driven involvement

The core of this view is that the quality of the interaction between patients and healthcare professionals especially influences patient involvement. ‘I had another internist before, but he didn't take me seriously. I noticed that this influenced my involvement: my motivation decreased. You do what you can but the relationship with the healthcare provider was damaged’ (Respondent Da 5). Patients perceived having trust in healthcare professionals (Statement 17, +4) and being taken seriously by them (Statement 16, +4) as most important. ‘The patient must be able to actively engage in a conversation with a healthcare provider. On the other hand, the healthcare provider must also allow that, of course’ (Respondent D20). In daily practice, this translates into patients valuing a good conversation with healthcare professionals (Statement 10, +3), encouragement of healthcare professionals to actively participate in decision‐making (Statement 23, +3) and the feeling that they can express their opinion (Statement 27, +3). Nevertheless, the personal characteristics of healthcare professionals such as ethnicity (Statement 34, −4) and gender (Statement 33, −4) are not considered important in this view, as in all the other views. Patients' own positive experiences with healthcare professionals (Statement 7, +2) are seen as relatively important, whereas the experience of other patients with active participation (Statement 42, −3) or having their own experience with active participation (Statement 5, −1) is seen as relatively unimportant.

### 3.5 View 3: Disease impact‐driven involvement

This view focuses on the impact of the disease on the patient, which provides intrinsic motivation to be involved. According to this view, the severity of the disease (Statement 32, +4) and its consequences for daily life (Statement 21, +4) are seen as most important for active patient involvement. *‘*The burden the patient carries by the impact on his daily life makes a patient motivated to do something about it. Then it will become important for you and you will feel the urge to do something about your healthcare’ (Respondent A6). Similarly, the patient's physical health condition (Statement 1, +3), belief in the advantages of active participation (Statement 18, +3) and intelligence (Statement 13, +3) are perceived as important. This view solely focuses on the patient level, and consequently, healthcare professionals' characteristics are perceived as least important (i.e., ethnicity, Statement 34, −4 and gender, Statement 33, −4). In line with this reasoning, other characteristics on the professional level are seen as less important, such as the collaboration between healthcare professionals (Statement 35, −3) and the continuity of care (Statement 39, −3) because ‘The health behaviour of a person isn't influenced by external factors but by internal motivation to do something with your disease. Every healthcare provider would motivate the patient, so it wouldn't matter if you changed healthcare provider’ (Respondent A6).

### 3.6 View 4: Cognition‐driven involvement

This view places considerable emphasis on the patient's understanding of what choices need to be made, the consequence of these choices and the relevance of these choices for their health. First, the patient's knowledge about the disease (Statement 3, +4) and the understanding of information about his/her own health (Statement 8, +3; Statement 16, +3) are seen as most important: ‘transferring information is important, it is important that everyone has all information’ (Respondent P12). In line with this, accessibility to one's own medical data (Statement 38, +2) and the ability to find information about the disease (Statement 9, +2) are perceived as relatively important. Underlying conditions for processing information are that patients feel taken seriously by healthcare professionals (Statement 16, +3) and the quality of the conversation between the patient and healthcare professionals (Statement 10, +2). ‘The ability to actively engage with healthcare providers about my disease and all the stuff that is relevant for my process is important. I want to have the opportunity to choose to be actively involved. It is the base for quality of care and active involvement of patients’ (Respondent A3). Second, the severity of the disease (Statement 32, +4) and its effect on daily life (Statement 21, +3) are seen as most important for active patient involvement. In line with this, the present physical health (Statement 22, +2) and the future expectations for the patient (Statement 1, +2) are perceived as relatively important for active participation. Also, here, the personal characteristics of the healthcare professional, ethnicity (Statement 34, −4) and gender (Statement 33, −4) are seen as least important. ‘A man or a woman, I don't care as long as they do it right’ (Respondent A12). It is notable that patients' personal characteristics that could influence the ability to process information are not considered as important for patient involvement, such as how well a patient masters the Dutch language (Statement 14, −3), their intelligence (Statement 13, −3) or their psychological health (Statement 2, −3).

## 4 DISCUSSION

Modern healthcare increasingly calls for patients to be actively involved in their healthcare process. Especially as care is increasingly more organized around the patient in a holistic manner, the patient becomes the linking pin in a dynamic network of healthcare professionals from different organizations. Some even consider patients as being part of the healthcare team in which care is basically coproduced.^19^, ^20^ This study offers insights into the diversity in perspectives among patients with a chronic disease on what is important to be actively involved in decision‐making about the content and process of treatment. This study may help to focus the efforts to enhance patient involvement. We identified four distinct views, which can be briefly summarized as follows: (1) *Enabled involvement*; these patients report that active involvement is influenced by the extent to which they are facilitated and empowered to be involved. (2) *Relationship‐driven involvement*; these patients state that active involvement mostly depends on the quality of the interaction and the relationship between patients and healthcare professionals. (3) *Disease impact‐driven involvement*; according to this view, involvement is based on the impact of the disease on the patient and the expected gains from being involved, creating intrinsic motivation. (4) *Cognition‐driven involvement*; this view is about the patient's understanding of the disease and the choices that need to be made.

These views provide a different perspective on factors influencing patient involvement than is usually presented in the literature that provides overviews of relevant factors. Often, factors are listed and categorized based on their intrinsic similarities, for example, patient‐related factors, contextual factors and so forth.^12^, ^15^ However, such lists seem to suggest that these factors have similar values for each patient. Furthermore, because of the number of factors involved, these categorizations may provide little support in prioritizing efforts to increase patient involvement. Finally, such lists neglect to show that factors from different categories also interact. For example, there may be considerable information available about a disease, but if a patient is not able to read or understand this information, it becomes irrelevant for the involvement of this patient. The views identified in this study show that not each factor is as important for each type of patient. It is not about optimizing each factor, but about creating the right conditions that fit the views and characteristics of specific groups of patients. For example, many studies discuss patient portals as a tool to better involve patients and stimulate self‐management.^50^ Patient portals are secure internet‐based platforms that offer patients the ability to view their personal health information. In practice, these portals are, however, only used by a select group of patients.^50^ One could hypothesize that differences in use may be partly explained by the views that we identified. Patients with a cognitive‐driven involvement are probably active users of portals, while patients with a relationship‐driven involvement may only use the portal if it is embedded in a positive relationship with healthcare professionals in which they are stimulated to use the portal and the portal itself facilitates interactions with the care provider. In line with this reasoning, one could also hypothesize that depending on the identified views, patient‐reported outcome measures (PROMs) will facilitate patient involvement in treatment decisions. It is likely that especially patients with an enabled involvement or a relationship‐driven involvement view will benefit from PROMS.

It is difficult to relate our findings to other studies that differentiate between views on patient involvement as these often focus on other aspects; for example, Jedeloo et al.^27^ specifically looked at patient preference for the level of involvement. However, the findings of van Hooft et al.^24^ can be related to our findings. They identified different views of nurses on how to support self‐management, which they named the gatekeeper, the clinician, the coach and the educator. The gatekeeper does not seem focused on patient involvement in decision‐making, and therefore, is not relevant for our comparison. The clinician is more inclined to involve the patient, but is very goal orientated and has a strong focus on adherence by monitoring and giving advice. This rational approach seems to fit best with a disease impact view of patients, but may provide too little room to codecide for patients with a cognitive view and is too little focused on the relationship for the other patient views. For the educator, professional knowledge is more important than patient experience as he or she focuses strongly on the illness and not on other life aspects. His or her aim is to provide the patient the necessary information and knowledge about his or her condition, so that he or she can make informed decisions. This perspective seems to relate strongly to the cognitive view of patients that we identified and also (although to a lesser degree) to the disease impact‐driven view, as they both have a primarily rational disease‐related perspective. The coach fits especially well with the enabled and relational‐driven view of patients, as this perspective is very much about the value of the relationship with the patient and seeing patients as partners in supporting them in living a ‘good life’. Therefore, although some of the nurse perspectives fit specific patient views, none seem to fit all; this suggests that nurses may need to be more flexible in their approach to stimulate patient involvement best.

From the patient perspective, this study thus shows that there is not a one‐size‐fits‐all approach to involving patients more actively in their healthcare journey. From the healthcare professional perspective, previous studies have shown heterogeneity in their approach towards patient involvement as well.^24^, ^25^ Managers and policy‐makers in the healthcare sector who aim to increase patient involvement should therefore be aware that views on patient involvement can differ on both sides of the table. Hibbard et al.^51^ developed a measure to assess professional beliefs about patient self‐management. Future studies should focus on how views of patients and healthcare professionals on patient involvement can be better aligned. Tools have also been developed to enhance patient involvement, such as patient portals. However, it is not clear if such tools sufficiently match the needs of patients.

## 5 LIMITATIONS AND STRENGTHS

Q‐methodology is well suited for exploring and describing the variety of views on a topic in a certain population in depth, but is less suited for making claims about the distribution of these views within the larger population or their associations with the characteristics of the population. This study thus informs about the key factors for active patient involvement in treatment decision‐making from the patient's perspective, but does not inform about which and how many patients with chronic conditions attach importance to these factors. For this purpose, survey methods could be applied in representative samples, asking respondents for their affinity with each of the views.^52^ Examples of such studies include Mason et al.^53^ and Mason et al.^54^ In addition, although the statement set was carefully developed for the purpose of this study based on the available literature on the topic, was thoroughly pilot‐tested and the main study did not provide indications that relevant factors for patient involvement were missing, the statement set may not be directly applicable in different healthcare settings and systems. Furthermore, despite the sizeable sample of respondents, the convenience sampling strategy and the choice for the three specific conditions might still have resulted in a selection bias, leading to certain views among patients with a chronic condition not being represented in this study. Future studies in patients with other chronic diseases, or a survey including these views in a larger sample of patients with chronic diseases,^52^ are needed to confirm this. Research could aim to recruit a more representative group of people with one or multiple chronic conditions by sampling respondents in terms of, for example, multiple chronic conditions, age, gender, level of education, type of and experience with the condition, health and social care setting and physical and mental state. Finally, Q‐methodology is not the appropriate method to make claims about the distribution of views within a sample or relate views to specific characteristics of patients, although this may be very relevant for practice, for example, to better understand the needs and wishes of people with digital and health literacy concerns. Future research should therefore either focus on replicating this study in such specific populations or use different methodologies, like surveys,^52^ to identify which patient characteristics relate to which views.

## 6 CONCLUSION

Patient participation depends on a ‘complex interplay of personal, physician and contextual factors’.^55^ This study showed four distinct views on what is important for active involvement in treatment decision‐making among patients with certain chronic conditions. However, it is still unclear which factors would be considered important in other patient groups, different health and social care settings and also in different cultural environments. Future research should continue to empirically explore the facilitators and barriers to patient involvement in healthcare teams, extending the theoretical understanding and sampling broadly to increase the generalizability of the findings.

## CONFLICT OF INTERESTS

The authors declare that there are no conflict of interests.

## AUTHOR CONTRIBUTIONS

Martina Buljac‐Samardzic, Mark A. Clark and Jeroen D. H. van Wijngaarden designed the study. Jeroen D. H. van Wijngaarden coordinated and collected the data together with students. N. Job. A. van Exel performed the data analyses. All authors contributed towards the interpretation of the data. Martina Buljac‐Samardzic wrote the first draft of the article, and all the authors provided feedback and approved the final manuscript.

## Data Availability

The data that support the findings of this study are available from the corresponding author upon reasonable request.
